# Risk factors and prediction model for necrotizing enterocolitis in preterm infants with gestational age ≤ 32 weeks: a retrospective cohort study

**DOI:** 10.3389/fped.2026.1792364

**Published:** 2026-06-02

**Authors:** Hao Li, GuiXiang Zeng, YaoXun Wu, Lin Cheng, LianFang Jing, HanLu Zhong, Yu Xie, Zhe Wei, KeMou Wu, Jun Fu, XiuLi Deng, HuaBo Tang, RenZhuang Huang, YanTing Lin, MingJie Lin, ShaSha Wei, DongMei Jiang, Yan Li

**Affiliations:** 1 RuiKang Clinical Medical College, Guangxi University of Chinese Medicine, Nanning, China; 2 Children’s Emergency Department, Maternity and Child Health Hospital of Guangxi Zhuang Autonomous Region, Nanning, China; 3 Department of Neonatology, Nanning Maternity and Child Health Hospital, Nanning, China; 4 Department of Neonatology, Women and Children’s Health Hospital of Yulin, Guangxi, China; 5 Department of Neonatology, Liuzhou Worker’s Hospital, Liuzhou, China; 6 Neonatal Medical Centre, Maternity and Child Health Hospital of Guangxi Zhuang Autonomous Region, Nanning, China; 7 Paediatric Neurology, Maternity and Child Health Hospital of Guangxi Zhuang Autonomous Region, Nanning, China

**Keywords:** necrotizing enterocolitis, prediction model, premature infants, retrospective cohort study, risk factors

## Abstract

**Objective:**

To develop a reference tool for necrotizing enterocolitis(NEC) prevention and treatment by constructing a predictive model for NEC risk in preterm infants (≤32 weeks’ gestation) in Guangxi, China.

**Methods:**

The clinical data of 497 preterm infants with gestational age ≤32 weeks managed at four neonatal care centers in Guangxi between January 2,019 and December 2021 were retrospectively reviewed. The cohort was randomly divided into a training set (for model development) and a test set (for model validation) in an 8:2 ratio. Within the training set, non-NEC infants were randomly selected to match NEC infants at a 1:1 ratio for comparative analysis. Univariate analysis was first performed to compare clinical indicators between the NEC and non-NEC groups and to identify potential predictors. Subsequently, independent risk factors were determined using binary logistic regression analysis, and a nomogram for predicting NEC risk was constructed using R software. Model performance was evaluated using the area under the receiver operating characteristic (ROC) curve, the Hosmer-Lemeshow goodness-of-fit test, and calibration curves.

**Results:**

The incidence of NEC among preterm infants with gestational age ≤32 weeks was 12.27% (61/497). Univariate analysis revealed significant differences between the two groups in gestational age, birth weight, 5-minute Apgar score, presence of neonatal respiratory distress syndrome (NRDS), intrauterine growth restriction (IUGR), sepsis, fungal infection, and the use of both invasive and non-invasive ventilation (*P* < 0.05). Multivariate logistic regression analysis identified IUGR (OR = 30.586), NRDS (OR = 22.955), sepsis (OR = 36.495), and invasive ventilator use (OR = 1.295) as independent risk factors for NEC (*P* < 0.05). A higher 5-minute Apgar score was identified as a protective factor (*P* < 0.05), indicating a decreased risk of NEC with increasing scores. Based on these factors, a nomogram prediction model was constructed using R software. The model demonstrated excellent discriminatory ability, with an area under the ROC curve (AUC) of 0.917 in the training set and 0.906 in the test set. The Hosmer–Lemeshow goodness-of-fit test for the test set (*χ*^2^ = 3.761, *P* = 0.807) indicated no significant difference between predicted and observed probabilities, suggesting good model calibration. The calibration curve approaches the 45-degree line, demonstrating good consistency between the model's predicted values and actual values.

**Conclusion:**

The predictive model developed in this study demonstrates good discriminatory power and calibration, and is effective in assessing the risk of NEC in preterm infants with a gestational age of ≤32 weeks in the Guangxi region. It provides valuable guidance for the early prevention and treatment of NEC in this population.

## 1 Introduction

Necrotizing enterocolitis (NEC) represents a devastating gastrointestinal disorder characterized by intestinal inflammation and necrosis, and is associated with substantial morbidity and mortality rates (1). This condition predominantly affects preterm and low-birth-weight neonates. The prevalence is approximately 1‰ among live births, escalating to 11% in very low-birth-weight infants (<1500 g) and reaching as high as 22% in extremely low-birth-weight infants (<1000 g) (2). Notably, the incidence of NEC demonstrates an inverse correlation with gestational age (3), with approximately 85% of cases occurring in preterm infants delivered before 32 weeks of gestation (4). The overall mortality rate for neonates diagnosed with NEC is 23.5%, potentially increasing to 50% in severe cases (2). Data from South Africa indicates that the mortality rate for infants undergoing surgery ranges from 48% to 50% (3). Even among survivors, the long-term prognosis remains guarded. The most serious sequelae include neurodevelopmental impairment and intestinal failure, with reported incidences of 24.8% and 15.2%, respectively. Among infants requiring surgical intervention for NEC, these rates increase dramatically to 59.3% for neurodevelopmental disorders and 35.3% for intestinal failure (2). Consequently, NEC continues to pose a significant threat to the health and survival of newborns, particularly those born prematurely.

Due to limited understanding of the fundamental physiological processes underlying NEC, its precise etiology and pathogenesis remain unclear. However, it is widely recognized that NEC is a complex multifactorial disease (5). The medical literature has documented numerous risk factors for NEC, encompassing prematurity, low birth weight, Apgar score, patent ductus arteriosus, blood transfusion, sepsis, chorioamnionitis, mechanical ventilation, congenital heart disease, intrahepatic cholestasis of pregnancy, and respiratory distress syndrome (3, 6). However, among the multitude of identified risk factors, a consensus panel comprising 35 international experts achieved substantial agreement only on gestational age, birth weight, and feeding practices as definitive contributors (7). Therefore, identifying the most clinically significant risk factors for NEC and incorporating them into a visual prediction model holds considerable potential for guiding clinical prevention and therapeutic decision-making.

Although numerous studies have attempted to develop prediction models for NEC incidence, the majority are constrained by their single-center design. The present multicenter, retrospective investigation analyzed clinical data from preterm infants (gestational age ≤32 weeks) across the Guangxi region. This study aimed to identify significant risk factors for NEC within this specific gestational age cohort and to construct a nomogram prediction model based on these findings. The resulting predictive tool is intended to facilitate risk stratification and inform prevention strategies for this vulnerable population.

## 2 Methods

### 2.1 Study population

From January 2019 to December 2021, clinical data were collected from preterm infants with gestational age ≤32 weeks admitted to four neonatal care centers: Guangxi Zhuang Autonomous Region Maternal and Child Health Hospital, Guangxi Nanning Maternal and Child Health Hospital, Guangxi Yulin Maternal and Child Health Hospital, and Guangxi Liuzhou Worker's Hospital. Inclusion criteria were: (1) gestational age ≤32 weeks; (2) transfer to the neonatal department within 24 h after birth; and (3) improvement or cure at hospital discharge. Exclusion criteria were: (1) presence of severe congenital defects or digestive tract malformations; (2) discharge against medical advice or death; and (3) Patients with incomplete clinical data, primarily including: lack of key imaging or clinical records necessary for NEC diagnosis and Bell staging, or lack of data on core exposure factors and outcome measures relevant to the statistical analysis, such as feeding method and initiation time. After screening, a total of 497 cases were ultimately included. This retrospective study was conducted by reviewing infant medical records and was approved by the Medical Ethics Committee of the Maternal and Child Health Hospital of Guangxi Zhuang Autonomous Region. Given the retrospective study design, informed consent was waived.

### 2.2 Methodology

The 497 eligible samples were randomly divided into a training set (for model construction) and a test set (for model verification) in an 8:2 ratio. In order to simulate the real clinical environment and improve the generalization ability of the model, The test set remained unprocessed and was used solely for model validation. Due to the large difference between the number of NEC and non-NEC, in order to balance the category, the model can better learn the characteristics of NEC and improve the sensitivity of the model, within the training set, non-NEC infants were randomly selected in a 1:1 ratio to match the number of NEC infants for comparative analysis.

### 2.3 Data collection

Clinical information was collected from the electronic medical record system, Based on the clinical relevance of the NEC and the availability of fully documented clinical data, the clinical information collected includes: maternal age ≥35 years, assisted reproduction, premature rupture of membranes, chorioamnionitis, prenatal corticosteroid use, sex, gestational age, birth weight, 5-minute Apgar score, mode of delivery (vaginal delivery, cesarean section, or dystocia), and the presence of the following conditions: NRDS, bronchopulmonary dysplasia (BPD), patent ductus arteriosus (PDA), IUGR, intracranial hemorrhage (grade ≥3), hyperbilirubinemia, cholestasis, hypoglycemia, hypothyroidism, sepsis, retinopathy of prematurity, fungal infection, and anemia. Treatment data included invasive ventilator use, non-invasive ventilator use, and total duration of oxygen therapy. In this study, neonatal NEC was diagnosed based on clinical manifestations and radiological findings, and severity was classified according to the revised Bell criteria. NEC cases in this study refer to infants with Bell stage 2 or above.

### 2.4 Statistical analysis

Statistical analyses were performed using SPSS software (version 25.0). A two-sided *P*-value of <0.05 was considered statistically significant. The analysis proceeded in two main stages. First, univariate analyses were conducted to compare clinical indicators between the NEC and non-NEC groups. Categorical data were presented as number (percentage) and compared using the chi-square test. For continuous data, normality was assessed. Normally distributed data were expressed as mean ± standard deviation (SD) and compared using the independent Student's t-test. Non-normally distributed data were expressed as median with interquartile range [IQR; P25, P75] and compared using the Mann–Whitney U test. Second, variables showing an association with NEC (*P* < 0.05) in the univariate analyses were entered into a binary logistic regression model to identify independent risk factors and evaluate its multicollinearity.

Based on the results of the multivariate regression, a nomogram prediction model was constructed using R software (version 4.4.3). The discriminatory ability of the model was evaluated by generating the receiver operating characteristic (ROC) curve and calculating the area under the curve (AUC) using SPSS. Model calibration was assessed with the Hosmer–Lemeshow goodness-of-fit test. Using the rms package in R (version 4.4.3), plot the calibration curve to evaluate the model's calibration.

## 3 Results

### 3.1 Univariate analyses

Among the 497 enrolled preterm infants, 61 developed NEC, corresponding to an overall incidence rate of 12.27%. Univariate analysis identified statistically significant differences between the NEC and non-NEC groups with respect to gestational age, birth weight, 5-minute Apgar score, presence of NRDS, IUGR, sepsis, fungal infection, and utilization of invasive or non-invasive ventilatory support (*P* < 0.05). Detailed results are presented in Table 1.

**Table 1:** Univariate analysis.

Variable	NEC group (*n* = 51)	Non-NEC group (*n* = 51)	*χ*^2^/t value	*P* value
Perinatal factors [n(%)]				
Maternal age ≥35 years	18 (35.3)	17 (33.3)	0.043	0.835
Assisted reproduction	8 (15.7)	10 (19.6)	0.270	0.603
Premature rupture of membranes	22 (43.1)	24 (47.1)	0.158	0.691
Maternal chorioamnionitis	3 (5.9)	4 (7.8)	0.153	0.695
Prenatal corticosteroid use	38 (74.5)	41 (80.4)	0.505	0.477
Birth characteristics				
Male [n(%)]	32 (62.7)	27 (52.9)	1.005	0.316
Gestational age [x¯ ± S]	29.617 ± 1.635	30.233 ± 1.477	−1.996	0.049
Birth weight [x¯ ± S]	1,257.16 ± 302.088	1,400.70 ± 262.517	−2.547	0.012
5-min Apgar score [M(P25,P75)]	9 (9, 10)	10 (9, 10)	−3.727	<0.001
Delivery mode [n(%)]				
Vaginal delivery	28 (54.9)	20 (39.2)	2.519	0.113
Cesarean section	22 (43.1)	30 (58.8)	2.511	0.113
Dystocia	1 (2.0)	1 (2.0)	0.00	1.0
Comorbidities [n(%)]				
NRDS	42 (82.4)	16 (31.4)	27.019	<0.001
BPD	26 (51.0)	21 (41.2)	0.986	0.321
PDA	23 (45.1)	18 (35.3)	1.020	0.313
IUGR	8 (15.7)	2 (3.9)	3.991	0.046
Intracranial hemorrhage (≥grade 3)	4 (7.8)	2 (3.9)	0.708	0.400
Hyperbilirubinemia	38 (74.5)	45 (88.2)	3.169	0.075
Cholestasis	24 (47.1)	22 (43.1)	0.158	0.691
Hypoglycemia	11 (21.6)	7 (13.7)	1.079	0.299
Hypothyroidism	0 (0.0)	1 (2.0)	1.010	0.315
Sepsis	17 (33.3)	1 (2.0)	17.270	<0.001
Retinopathy of prematurity	8 (15.7)	5 (9.8)	0.793	0.373
Fungal infection	6 (11.8)	0 (0.0)	6.375	0.012
Anemia	46 (90.2)	43 (84.3)	0.793	0.373
Treatment [M(P25,P75)]				
Invasive ventilator (days)	1 (0, 5)	0 (0, 1)	−3.268	0.001
Non-invasive ventilator (days)	7 (3.25, 20.75)	5 (3, 14)	−2.237	0.025
Total oxygen therapy (days)	22.5 (8.5, 35.75)	20 (9, 35)	−0.569	0.569

NRDS, neonatal respiratory distress syndrome; BPD, bronchopulmonary dysplasia; PDA, patent ductus arteriosus; IUGR, intrauterine growth restriction.

### 3.2 Multivariate regression analysis

Multivariate logistic regression analysis was subsequently performed incorporating all variables that demonstrated statistical significance in the univariate analysis. The results indicated that IUGR, NRDS, sepsis, and invasive ventilator use were independently associated with increased NEC risk (*P* < 0.05). In contrast, the 5-minute Apgar score emerged as a significant protective factor (*P* < 0.05), with higher scores conferring reduced NEC risk. The results of the multicollinearity assessment for the above factors show that the VIF value for NRDS is 1.188, for IUGR it is 1.093, for sepsis it is 1.189, for invasive mechanical ventilation it is 1.092, and for the 5-minute Apgar score it is 1.208, indicating that there is no high correlation among these predictor variables. Complete results of the multivariate analysis are summarized in Table 2.

**Table 2:** Multivariate logistic regression analysis.

Variable	B	SE	Wald χ^2^	*p* value	OR	95% CI	VIF
Gestational age	0.196	0.324	0.367	0.544	1.217	0.645–2.298	-
Birth weight	0.000	0.002	0.079	0.779	1.000	0.996–1.003	-
5-minute Apgar score	−1.711	0.591	8.388	0.004	0.181	0.057–0.575	1.208
NRDS	3.134	0.812	14.883	<0.001	22.955	4.672–112.789	1.188
Fungal infection	22.185	12,136.310	0.000	0.999	4314628443	0.000–	-
IUGR	3.421	1.189	8.282	0.004	30.586	2.977–314.235	1.093
Neonatal sepsis	3.597	1.331	7.307	0.007	36.495	2.689–495.388	1.189
Invasive ventilator	0.258	0.122	4.479	0.034	1.295	1.019–1.645	1.092
Non-invasive ventilator	−0.065	0.041	2.459	0.117	0.937	0.864–1.016	-
Constant	8.114	9.810	0.684	0.408	3,339.523		

NRDS, neonatal respiratory distress syndrome; IUGR, intrauterine growth restriction.

### 3.3 Development of a nomogram prediction model

**Figure 1:**
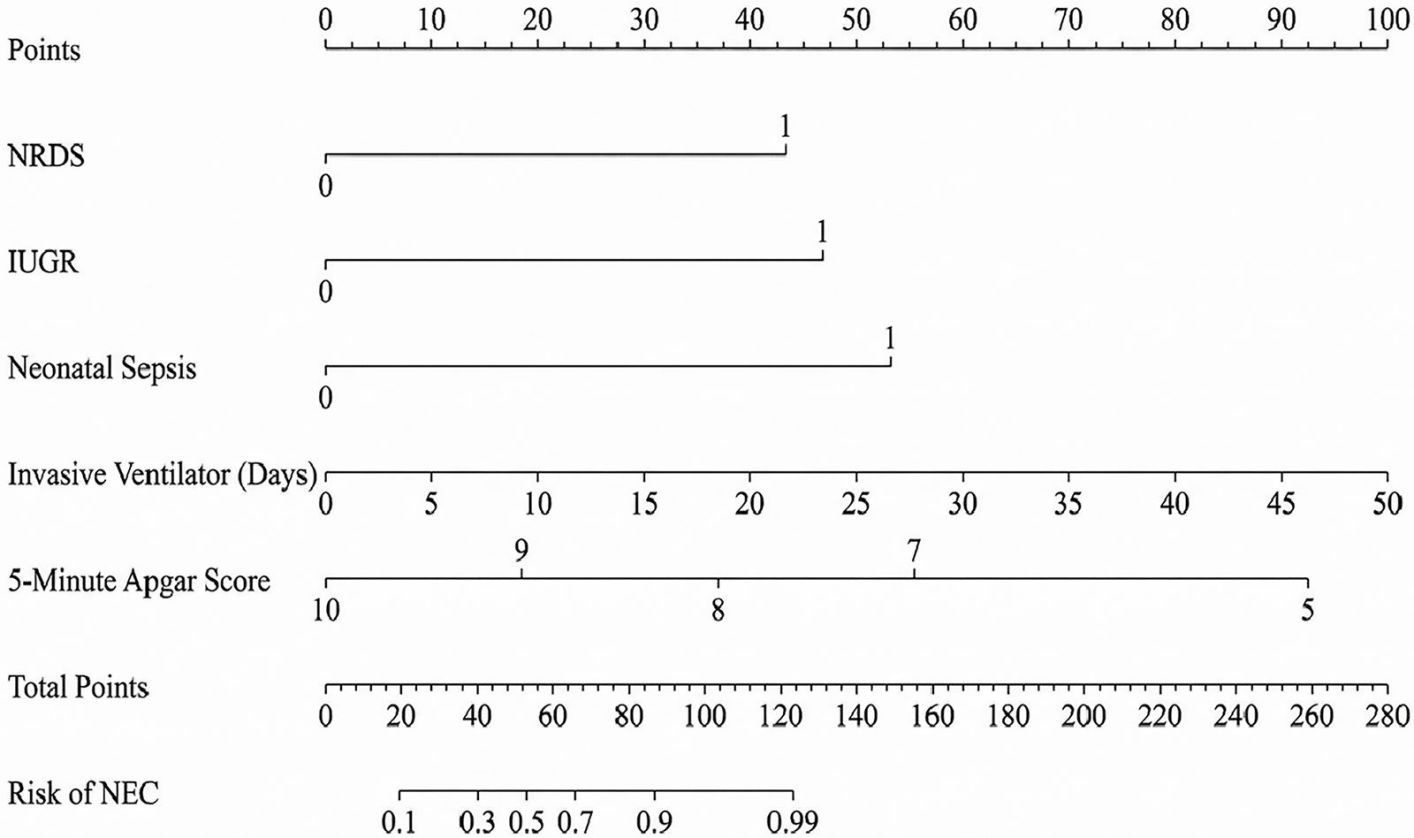
Nomogram prediction model for NEC risk in preterm infants (≤32 weeks' gestation).

Utilizing the results of the multivariate logistic regression analysis, a nomogram prediction model was constructed using R software (version 4.4.3). The model incorporated five variables: NRDS, IUGR, sepsis, invasive ventilation use, and 5-minute Apgar score (Figure 1). The variable codes are shown in Table 3, and the complete regression equation is: logit(P)= 8.64 + 2.836[NRDS (1)] + 3.071[IUGR (1)] + 3.49[Sepsis (1)] + 0.131(`Invasive mechanical ventilation`) - 1.212(`5-minute Apgar score `).

**Table 3:** Variable coding.

Variable	Coding
NEC (outcome)	0 = no (Bell stage < II), 1 = yes (Bell stage ≥ II)
5-minute Apgar score	Continuous (range 0–10), entered as continuous variable
NRDS	0 = no, 1 = yes
IUGR	0 = no, 1 = yes
Sepsis	0 = no, 1 = yes
Invasive mechanical ventilation	Continuous (days of use), entered as continuous variable

### 3.4 Model reporting standards and interpretation

The model is presented as a bar chart (see Figure 1), and the reported result is a percentage representing the probability of developing the condition. Specifically: R software assigns scores to independent risk factors based on their odds ratios (OR). NRDS scores 43 points, IUGR scores 46 points, and sepsis scores 53 points, as indicated by the value corresponding to the code “1” in the figure. The use of invasive mechanical ventilation is assigned a score based on the number of days; if not used, the score is 0. The 5-minute Apgar score is a protective factor: a higher score results in a lower risk score, while a lower score results in a higher risk score, with specific scores corresponding to each value. When applying the model, based on the specific circumstances of preterm infants with a gestational age ≤32 weeks, the scores corresponding to their existing risk factors are summed to obtain a total score. The value on the risk axis that corresponds vertically to this total score represents the probability of NEC. The higher the total score, the larger the corresponding percentage, indicating a higher probability of NEC.

### 3.5 Nomogram evaluation

**Figure 2:**
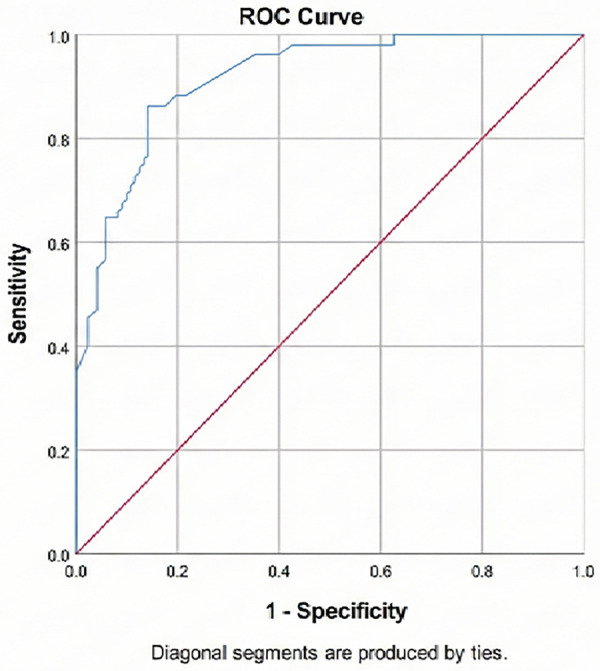
ROC curve training set.

**Figure 3:**
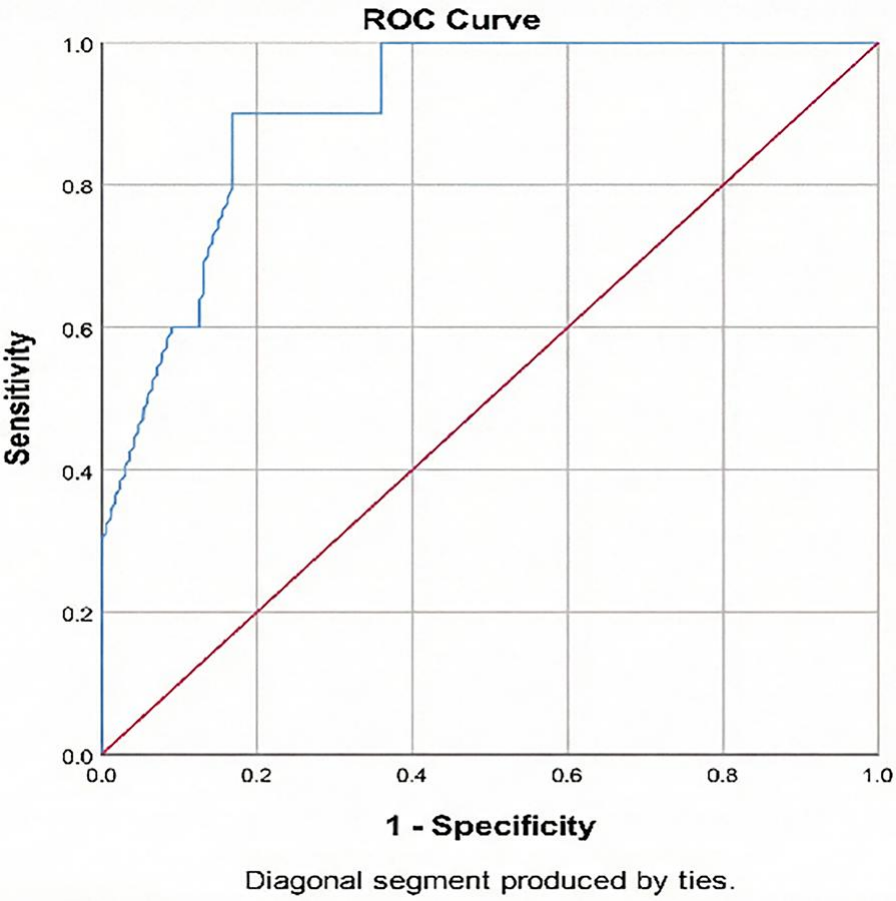
ROC Curve testing set.

**Figure 4:**
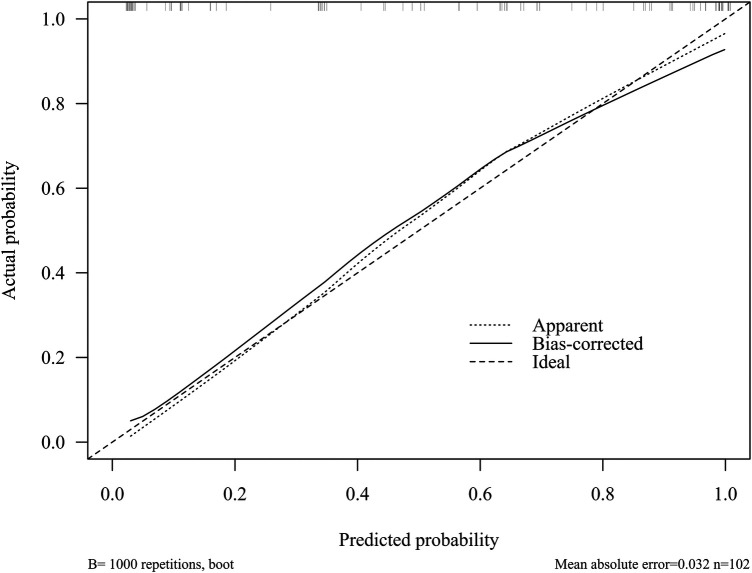
Calibration curves-training set.

**Figure 5:**
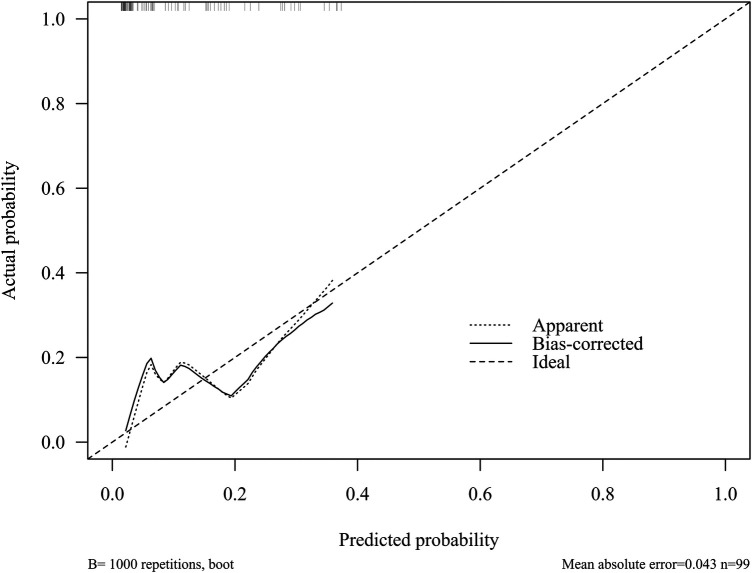
Calibration curves-testing set.

We used SPSS 25.0 software to plot ROC curves for the line-plot model and calculate the AUC. The results showed that the AUC for the training set was 0.917 (95% CI: 0.865–0.969), as shown in Figure 2, and the AUC for the test set was 0.906 (95% CI: 0.828–0.983), as shown in Figure 3, indicating that the model has good discriminatory power. The Hosmer-Lemeshow goodness-of-fit test yielded *χ*^2^ = 3.761 and *P* = 0.807, indicating that there is no significant difference between the model's predicted probabilities and the observed probabilities, and that the model has good calibration. Using the rms package in R (version 4.4.3), calibration curves were plotted for the training set (Figure 4) and the test set (Figure 5). The calibration curves tend toward the ideal 45-degree line across most risk intervals, indicating good calibration performance.

### 3.5 Comparison with similar predictive models

This model is one of the few developed using multicenter data from China, and its AUC value exceeds 0.90, outperforming existing models (which typically range from 0.75 to 0.85).

## 4 Discussion

Necrotizing enterocolitis (NEC) represents a severe neonatal inflammatory bowel disease and constitutes one of the most prevalent gastrointestinal emergencies encountered in the neonatal period. Although approximately 10% of cases occur in full-term infants, preterm neonates account for the overwhelming majority (90%) of all NEC diagnoses. The reported incidence ranges from 1% to 5% among neonates admitted to intensive care units, with prevalence rates of 7%–14% and mortality rates approaching 20%–50% observed in very low birth weight (VLBW; 500–1500 g) infants. As such, NEC remains a formidable clinical challenge (5). Given that the underlying pathogenesis is not yet fully characterized, the identification of high-risk factors and their integration into visual predictive models carries substantial clinical significance for disease prevention and management. While domestic multicenter investigations have reported NEC incidence rates fluctuating between 3.9% and 10.7% in preterm infants born before 32 weeks’ gestation (8), the incidence observed in our study cohort was comparatively elevated. Considering the high mortality and unfavorable prognosis associated with NEC, systematic screening for high-risk factors is of paramount importance. Such screening enables timely clinical intervention, which may ultimately reduce disease incidence and improve patient outcomes. In the present investigation, we employed a machine learning methodology integrated with perinatal clinical factors to develop a comprehensive NEC prediction model. Our findings demonstrated that neonatal respiratory distress syndrome (NRDS), intrauterine growth restriction (IUGR), sepsis, and invasive ventilator use constituted key predictive factors for NEC in preterm infants. The model achieved an area under the receiver operating characteristic curve (AUC) of 0.906 and a Hosmer–Lemeshow (HL) test *P*-value of 0.807 on the test set, thereby confirming its robust discriminative ability, satisfactory calibration, and favorable clinical interpretability.

Neonatal respiratory distress syndrome (NRDS), resulting from inadequate pulmonary surfactant synthesis, manifests clinically with tachypnea, expiratory grunting, intercostal retractions, and cyanosis. This condition represents a principal cause of hypoxemia and respiratory failure in the preterm population (9, 10). Hypoxia can elicit a “diving reflex” response, whereby blood flow is preferentially redistributed to the brain and heart at the expense of the splanchnic circulation, resulting in intestinal hypoperfusion or frank ischemia. Early pathogenic theories regarding NEC proposed ischemia as a primary or initiating event, hypothesizing that hypoxic-ischemic injury directly damages the neonatal intestinal mucosa (5). Such damage compromises intestinal barrier integrity, thereby facilitating bacterial translocation and triggering an inflammatory cascade that ultimately culminates in NEC development. Subsequent experimental animal studies have corroborated hypoxia as a critical driver of intestinal injury (11). More contemporary research has demonstrated that hypoxia can induce oxidative stress by disrupting the equilibrium between free radical generation and neutralization, leading to cellular oxidative damage. Indeed, NEC has been characterized as a classic manifestation of neonatal oxygen-free radical disease (12). The present study's identification of NRDS as a significant risk factor for NEC is concordant with the conclusions of a 2024 systematic review and meta-analysis examining factors influencing NEC in Chinese preterm infants (6).

Intrauterine growth restriction (IUGR), also known as fetal growth restriction (FGR), is defined as a condition in which a fetus fails to achieve its expected growth potential *in utero*. It is typically diagnosed when the fetal abdominal circumference (AC) or estimated fetal weight (EFW) falls below the 10th percentile for gestational age (13). A poor intrauterine environment can trigger a “brain-sparing effect,” whereby blood flow is preferentially redistributed to vital organs such as the brain and heart at the expense of other systems, including the gastrointestinal tract. This leads to intestinal hypoperfusion and chronic hypoxia in IUGR fetuses. Furthermore, this state is thought to impair the development and function of circulatory regulatory T-cell populations (14, 15). The consequent compromise in intestinal perfusion and immune regulation is believed to contribute to the pathogenesis of neonatal NEC. This study's finding that IUGR is a significant risk factor for NEC aligns with the results of a previous Chinese multicenter cohort study (16).

Sepsis, a serious systemic infection, is a well-established risk factor for NEC. Studies indicate that infants with sepsis have nearly triple the risk of developing NEC compared to those without (17). In preterm infants, the risk is compounded by gastrointestinal immaturity, characterized by an underdeveloped intestinal mucosal barrier and increased gut permeability, which facilitates bacterial translocation and infection (6). Once established, infection prompts the release of inflammatory mediators. This cascade triggers neutrophil activation, increases vascular permeability, and generates reactive oxygen species, leading to vasoconstriction and ischemia-reperfusion injury. The consequent damage to the mucosal barrier ultimately culminates in NEC (18). In the present study, sepsis was assigned 53 points in the predictive nomogram, underscoring its substantial weight as a risk factor.

This study identified the use of invasive mechanical ventilation as a significant risk factor for NEC, with both univariate and binary logistic regression analyses yielding statistically significant results (*P* < 0.05). This finding is supported by multiple domestic and international studies reporting mechanical ventilation as a risk factor for NEC (6, 19, 20). Furthermore, research indicates a positive correlation between the duration of mechanical ventilation and NEC risk, with longer ventilation periods associated with increased incidence (21). Specifically, ventilation exceeding 60.5 h has been identified as an important risk factor for NEC in very low birth weight (VLBW) infants (22). Although the precise mechanisms linking mechanical ventilation to NEC remain underexplored, hypoxia, infection, and inflammatory responses are hypothesized to play mediating roles. Infants requiring mechanical ventilation often present with respiratory failure or critical illness, conditions frequently complicated by infection. The interplay of hypoxia, infection, and systemic inflammation in these patients is thought to promote the development of NEC (22).

The Apgar scoring system, developed in the 1950s by anesthesiologist Virginia Apgar, is a standard tool for assessing neonatal transition after birth and determining the need for intervention (23). Research by Feng et al. indicates that a 5-minute Apgar score between 0 and 7 constitutes a significant risk factor for NEC, with lower scores correlating with higher NEC incidence—a relationship attributed to perinatal asphyxia and resultant hypoxia (7). The pathophysiological essence of asphyxia is tissue hypoxia, which in newborns can trigger a compensatory “diving reflex.” This hemodynamic response shunts blood away from the mesentery, reducing intestinal perfusion and potentially leading to mucosal ischemia and necrosis, thereby contributing to NEC pathogenesis. Globally, asphyxia accounts for approximately 20% of neonatal deaths, a proportion that rises to 60% among preterm infants; notably, 6% of these cases may progress to NEC (7). In contrast to the established risk associated with low scores, this study identified a 5-minute Apgar score of 10 as a protective factor against NEC.

Gestational age and birth weight are well-established, closely related risk factors for NEC (2, 3, 8). The incidence of NEC exhibits a strong inverse correlation with both parameters. For instance, incidence ranges from 3.9% to 10.7% in infants with gestational age below 32 weeks and from 5% to 10% in very low birth weight (VLBW) infants (24). More specifically, incidence reaches approximately 12% in infants weighing 501–750 grams, with an estimated 3% decrease in risk for every 250-gram increase in birth weight. Preterm birth is a principal risk factor for NEC, primarily due to the immaturity of the intestinal mucosal barrier and immune response. The intestinal epithelium—composed of enterocytes, tight junctions, goblet cells, and pattern recognition receptors—forms a critical physical barrier. In preterm infants, immature enterocytes compromise tight junction integrity, leading to increased gut permeability and facilitating the invasion and translocation of bacteria, particularly Gram-negative species such as Escherichia coli. Concurrently, a reduced number of goblet cells results in diminished mucus production, leaving the vulnerable epithelium exposed to luminal pathogens and toxins. Toll-like receptors (TLRs), especially TLR4, are key mediators in NEC pathogenesis. TLR4 expression is elevated in the preterm intestine. Colonizing Gram-negative bacteria activate TLR4 on the intestinal epithelium, triggering a cascade of detrimental effects: increased enterocyte apoptosis, impaired mucosal repair, and the release of pro-inflammatory cytokines. Furthermore, bacterial translocation can activate TLR4 on mesenteric endothelial cells, leading to reduced intestinal blood flow and contributing to ischemic necrosis. The characteristics of preterm infants susceptible to NEC also include the immaturity of other physiological processes, such as reduced digestion and absorption of nutrients and impaired intestinal motility. These factors have been confirmed in humans with NEC and in experimental animal models of the disease (25, 26). Consistent with the literature, this study confirmed gestational age and birth weight as significant risk factors for NEC. However, multivariate analysis indicated that they were not independent risk factors in our predictive model.

In summary, this study employed machine learning methods to develop a predictive model for NEC in preterm infants born before 32 weeks of gestation. We identified that NRDS, IUGR, sepsis, mechanical ventilation, and the 5-minute Apgar score are predictive indicators of NEC. Internal validation demonstrated that this predictive model has good reliability. This provides clinicians with a simple and intuitive tool for predicting NEC, which is helpful for early detection and early intervention, reducing the incidence of NEC, and improving the quality of life in this population. Limitations of this study: 1. There are limitations in data processing and feature selection; directly excluding missing values and screening variables based on single factors may introduce bias. Future studies will apply multiple interpolation methods to reduce information loss and utilize the LASSO regularization method to mitigate overfitting. 2. This study did not use a complete cohort to build the model, limiting its stability. We will further validate our results by increasing the number of cases and conducting a study using a complete cohort. 3. The validation approach is limited; currently, only internal validation has been completed. External validation using a multicenter independent cohort is still required to ensure reliability for clinical application. 4. The inclusion of variables is not comprehensive; due to limitations of retrospective records, the model lacks important variables related to feeding and enteral nutrition. Although the current objective variables make the model more practical in resource-limited settings such as primary care, future efforts should involve prospective, standardized data collection to optimize the model.

## Data Availability

The raw data supporting the conclusions of this article will be made available by the authors, without undue reservation.
